# The Role of Sociodemographic and Psychological Variables on Risk Perception of the Flu

**DOI:** 10.1177/2158244017718890

**Published:** 2017-07-14

**Authors:** Elena Commodari

**Affiliations:** 1 University of Catania, Italy

**Keywords:** risk perception, infectious diseases, self-efficacy, personality, education

## Abstract

Influenza is a source of mortality and morbidity, and vigilance of health authorities for flu viruses is high. The World Health Organization (WHO) highlighted that the first lines of defense against infectious diseases are behavioral, and risk perception affects behavioral measures. This study investigated risk perception of influenza and the role of sociodemographic and psychological variables on perceived risk. Participants were 442 adults. The research was conducted using three measures: an adjustment of the “Risk Perception of Infectious Disease Questionnaire,” the “General Self-Efficacy Scale,” and the short form of the “Italian Personality Inventory.” The results showed that age, education, self-efficacy, and personality influenced risk perception. The evidence that sociodemographic and psychological factors contribute to risk perception of a disease shows the need to take into account these variables in the planning of informative campaigns, with the aim to achieve favorable changes in public behavior. These issues might have implications for the ameliorating health communication efforts and successful response to new influenza outbreaks.

## Introduction

### Influenza and Health Services Guidance

Influenza is a source of morbidity and mortality, with impacts on national health care systems and economic consequences (Giannattasio et al., 2015; World Health Organization [WHO], 2015). In recent decades, the bird flu and the 2009 pandemic influenza A/H1N1 showed people the potential risks connected to this disease. The emergence of a new influenza virus to which many people had no preexisting immunity characterized the 2009 pandemic.

Pandemic influenza was a mild disease in many individuals. However, it affected the younger population more than previous flu viruses and produced a larger than expected number of severe or fatal cases in pregnant women, obese people, and healthy people (Louie et al., 2009).

From the pandemic, the A/H1N1 virus circulates as a seasonal virus, and vaccination contributed to control its diffusion. However, in several countries such as in Italy, the vaccinal coverage for flu is low (Ministry of Health, Italy, 2016). Moreover, although the actual context is different from the pandemic context, the A/H1N1 virus continues to present some characteristics that differentiate it from other flu viruses. For this reason, although media attention for this virus decreased in the past years, the vigilance of health authorities remains high.

A/H1N1 virus has continued to cause severe cases in the recent years, especially in healthy middle adults and pregnant women (e.g., Chowell et al., 2012; Mishra, 2015). In this regard, the European Centre for Disease Prevention and Control (ECDC; 2014) reported high circulation of A/H1N1 virus during 2014. A recrudescent wave of this virus was reported in Mexico in winter 2013 to 2014, and Davila and colleagues (Dávila et al., 2014) documented an increased number of A/H1N1-related hospitalization and death. In particular, they reported a high proportion of individuals added 30 to 59 years hospitalized with A/H1N1. Similarly, Mishra (2015) reported that in winter 2014 to 2015, pandemic A/H1N1 virus determined a higher number of cases and deaths than those of the previous flu seasons in India, and WHO (2015) described a widespread activity of the pandemic virus during the first weeks of the 2015 flu season in Italy. In particular, according to the Italian National Surveillance System of Flu (Influnet), the A/H1N1 virus was the most diffuse flu virus during the first 6 weeks of the epidemic in Italy, and it was the dominant virus in the majority of the severe cases. Serious cases and deaths were reported among pregnant women in several regions of Italy, such as Emilia Romagna (General Direction of Health, Social, and Integration Politics of Emilia Romagna, 2015). In this regard, the local health offices in Italy recommended the need to continue to consider pregnant women at high risk for this type of virus and highlighted the necessity to increase the vaccinations for all the risk groups. Vaccination is, in fact, the most effective mean of mitigating the harmful health care and social effects of influenza. However, despite the availability of flu vaccines, the Italian public’s trust in vaccination has declined in the last few years, and influenza vaccination rate has decreased among at-risk adults (Gasparini, Amicizia, & Panatto, 2016).

From the pandemic, WHO issued guidance on recommended preventive activities for people with the aim to control the diffusion of flu viruses. The 2010 guidance (WHO, 2010) highlighted that behavior of individuals influenced outbreaks of influenza and stressed the importance for people to take steps to protect themselves through protective actions. In agreement with that guidance, ECDC (2011) reported that the first lines of defense during a pandemic are behavioral. ECDC (2011) highlighted that knowledge influences behavior in what concerns the perceived risk and health beliefs.

The Pandemic Influenza Risk Management–WHO Interim Guidance (WHO, 2013) emphasized this concept. It evidenced the need to develop strategies to communicate with individuals to improve their ability to take appropriate actions before, during, and after a pandemic. Moreover, WHO (2013) highlighted the need to assess the ability of the communication channel to reach all target population groups and to develop mechanisms that guarantee the widest circulation of information.

### Psychological Variables and Infectious Diseases

People beliefs contribute to control the diffusion of infectious diseases, and the classic health belief model considers risk perception as one of the key drivers of health behavior (Brewer, Weinstein, Cuite, & Herrington, 2004; Ibuka, Chapman, Meyers, Li, & Galvani, 2010). Risk perception influences precautionary actions (Brewer et al., 2007; J. H. Jones & Salathe, 2009; Lau, Griffiths, Choi, & Tsui, 2010; Rubin, Amlot, Page, & Wessely, 2009), and the literature on severe acute respiratory syndrome suggests that people are more likely to comply with health-related recommendations if they perceive high probability to get a disease (Tang &Wong, 2003).

The health belief model was originally formulated to model the adoption of preventive health behaviors in the United States (Rosenstock, 1974). The underlying concept of this model is that personal beliefs or perceptions determine health behavior. This model describes six constructs that predict health behavior (C. L. Jones et al., 2015): perceived seriousness, perceived susceptibility, benefits to action, barriers to action, cues to action, and self-efficacy. “Seriousness” and “susceptibility” concern the perception of risk for health. Perceived “seriousness,” also called severity, concerns the individual belief about the severity of a disease. It is an index of how a person thinks that a disease could be serious for him. Perceived “susceptibility,” also called vulnerability, concerns how a person considers himself at risk to have a disease. It comprises two dimensions. The first, “personal susceptibility,” is the perceived probability that one will be harmed by a hazard (Rogers, 1983). The second, “comparative susceptibility” is the perceived probability that a hazard will hurt one compared with other people of the same age and gender.

The third dimension, perceived “benefit,” involves the individual opinions of the value and usefulness of a new behavior in decreasing the risk of developing a disease. The fourth dimension, perceived “barriers” or obstacles concerns the evaluation of the impediments to the adoption of action or behavior that a person could execute to protect oneself.

In addition to this dimension, the fifth dimension, “cue to action,” involves all the variables that can move people to change their behavior, such as illness of a family member. Finally, “self-efficacy” concerns the confidence in the personal ability to perform preventive measure (Bandura, 1998).

Perceived self-efficacy regards the perception of the own ability to engage in protective actions. It is relevant for clinical practice and behavioral change (Ruiter, Abraham, & Kok, 2001). Self-efficacy was found as a predictor of the intention and practice of health behavior and risk perception (Joffe, 2003; Seyde, Taal, & Wiegman, 1990). A recent study of Cho and Lee (2015), which investigated the influence of self-efficacy and risk perception on behavioral intentions related to the A/H1N1 flu pandemic, showed that culture affected self-efficacy and risk perception. According to the authors, the way in which people perceive and respond to risks varied across nationalities and cultures. They found that self-efficacy and risk perception had stronger effects on behavioral intention in the American than in the Korean people.

Personality traits are another determinant of the perceived risk. Traits are the basic dimensions of personality (McCrae & Costa, 1987, 1997). Although numerous models (e.g., McCrae & Costa, 1997; Perussia, 2005) are developed, all researchers agree that traits contribute to the individual differences in cognitive, emotional, and behavioral functioning (e.g., Costa, Terracciano, & McCrae, 2001; McCrae & Costa, 1997; McCrae, Terracciano, & 78 Members of the Personality Profiles of Cultures Project, 2005).

Personality traits affect the perception of the events, influencing health. Individual differences in traits contribute to the responses to diseases (Hill, Turiano, Hurd, Mroczek, & Roberts, 2011). Moreover, personality facets were found to be predictive of risk perception compared with, or in association with age, gender, and educational level (Chauvin, Hermand, & Mullet, 2007).

Some authors (Jokela et al., 2013; Kern & Friedman, 2011; Turiano, Chapman, & Mroczek, 2015) showed that the traits are related prospectively to **health** status in adulthood, and predict health behaviors and mortality risk. Conscientious individuals, for example, tend to engage in health-protective behaviors and avoid risky behavior (Bogg & Roberts, 2004; Friedman, 2008; Kern & Friedman, 2011). The trait of extroversion and conscientiousness was found to have a moderating effect on changes in dangerous behavior, such as smoking behavior, but risk perception was involved in the prediction of the outcomes (Hampson, Andrews, Barckley, Lichtenstein, & Lee, 2006). Other studies (e.g., Beier & Ackerman, 2003) found that happy and optimistic individuals engage in greater quantity and quality of pleasant activities than neurotic and pessimistic people. Furthermore, openness predicts healthy outcomes. In this regard, a study (Gaygısız, Gaygisiz, Ozkan, & Lajunen, 2012) on the behavioral reactions to the A/H1N1 virus during the later stage of the epidemic showed that personality factors moderately influenced the behavioral responses. The belief of the individuals in the effectiveness of their behavior was the primary factor that influenced the behavioral responses.

### Research Aims

Influenza is a problem of public health although the impact on the health of different flu viruses is variable. The health care systems pay attention to the diffusion of the seasonal flu viruses and highlight the importance of behavioral measures to control influenza. Moreover, they evidence the needs to individuate communication channel to favor the adoption of preventive behavior.

It is known that one of the main variables that affect behavioral measures is risk perception, and its surveillance could contribute to ameliorate health risk communication, increasing successful changes in behavior. Despite this, risk perception for the potentially dangerous viruses was not largely examined.

Based on these considerations, the present study investigated risk perception for the A/H1N1 virus, during the postpandemic period in Italy, before the 2015/2016 flu season. Three dimensions of risk perception were investigated: perceived “seriousness,” “personal susceptibility,” and “comparative susceptibility.” Moreover, the role of sociodemographic, self-efficacy and personality on the perception of the risk related to this virus was also analyzed. The A/H1N1 virus circulates as seasonal virus and, although the actual context is different from those that characterized the pandemic, health authorities continue to pay attention to its diffusion because this virus presents some characteristics that differentiate it from the other seasonal flu viruses.

## Method

### Participants

Participants were 442 adults (age range = 19-70, 188 males, 254 females; see Table 1) who were in the waiting rooms of two general medicine ambulatories in a large town of Italy. All the 448 persons, who were in ambulatory during 7 working days, were invited to participate in the study. Four participants affected by chronic and severe pathologies and two who did not provide informed consent were excluded from the study. Of the remaining 442 participants, 238 were waiting for a medical consultation for mild or not serious pathologies or health-status certification release, 204 were companions. None of them were suffering from serious diseases and had been in a hospital for the past 8 months (see Table 1 for the sociodemographic characteristics of the sample). The choice to recruit adults who were in a medical waiting room, but were not affected by severe diseases, was derived from the intention to involve study persons who were not seriously ill but were potentially informed on the health measures for the common transmissible pathologies. The research was conducted before the start of the vaccinal campaign in Italy; for this reason, none of the participants was vaccinated against the flu. Research conforms to the Helsinki Declaration outlining the principles for research involving human subjects. Participants provided informed consent.

**Table 1.** Sociodemographic Distribution of the Participants (*N* = 442).

	*n*	%
Gender		
Men	202	42.5
Women	240	57.5
Age		
18-30	102	23.1
31-40	52	11.8
41-50	96	21.7
51-60	78	17.6
65 or older	114	25.8
Level of education		
Primary school	98	22.2
Secondary school	112	25.3
High secondary school	176	39.8
University degree	56	12.7
Household size		
1	30	6.8
2	110	24.9
3	60	13.6
4	138	31.2
5	100	22.6
6	4	0.9
Presence of children until age 12		
Yes	86	19.5
No	356	80.5

### Procedures

Data were collected 1 month before the start of the 2015/2016 flu season in Italy. Three psychologists administered the measures in a quiet room of the medical ambulatories (mean time of administration: 30 min).

### Measures

The research was conducted using three measures. The first measure was an adjustment of Risk Perception of Infectious Disease Questionnaire (Brug et al., 2004). It has been used in many previous studies (e.g., de Zwart et al., 2009; de Zwart, Veldhuijzen, Richardus, & Brug, 2010; Veldhuijzen, de Zwart, Voeten, & Brug, 2006) and translated into several languages (Veldhuijzen et al., 2006). In this version, the participants have to respond to several questions using 4-point Likert-type scales. The original 10-point Likert-type scales have been substituted with 4-point Likert-type scales because this type of recording of data is more common in Italy. The questionnaire comprised 85 items. The term “A/H1N1 influenza” substituted the word “SARS” of the original form.

The questionnaire collected sociodemographic information (sex, age, the size of household, education level, employment status, and household composition) and measured the perceived “seriousness,” “personal susceptibility,” and “comparative susceptibility” to some diseases (e.g., high blood pressure, diabetes, common cold, and others). Although the respondents completed all the parts of the questionnaire, only the responses that concerned the A/H1N1 flu were considered in this study. The participants were invited to report (a) how serious it would be for them to get the disease, (b) how likely they thought it would affect them, and (c) whether they would have a smaller or larger chance to get each of these diseases, compared with the other people of the same age and gender in Italy, in the forthcoming flu season.

The General Self-Efficacy Scale (GSE; Schwarzer & Jerusalem, 1995) evaluated self-efficacy. This 4-point Likert-type scale consisted of 10 items (global score from 10 to 40). The GSE is reliable (Cronbach’s alphas ranged from .76 to .90 in samples from 23 nations) and one-dimensional across cultures. Numerous correlation studies documented its criterion-related validity (Schwarzer & Jerusalem, 1995). Positive coefficients were found with positive emotions, dispositional optimism, and work satisfaction. Negative coefficients were found with depression, anxiety, stress, burnout, and health complaints (Schwarzer, Bäßler, Kwiatek, Schröder, & Zhang, 1997).

The short form of the Italia Personality Inventory (ITAPI-S; Perussia & Vaino, 1996) consisted of 28 items and measured seven personality traits (“dynamicity,” “susceptibility,” “empathy,” “conscientiousness,” “imagination,” “defensiveness,” and “introversion”). Some key concepts describe each trait. “Dynamicity” (reliability coefficient: Cronbach’s α = .86) concerns some psychological characteristics, such as attitude to take the initiative, curiosity, and liveliness. Dynamic people are enterprising and full of interests.

“Susceptibility” (reliability coefficient: Cronbach’s α = .86) concerns the attitude to discomfort, fear, and suffering. People with high scores in this trait often are sad and can change the mood easily. “Empathy” (reliability coefficient: Cronbach’s α = .79) comprises solidarity, sociability, and sensitivity. The empathic people can recognize the emotions of other people and understand their feelings. “Conscientiousness” (reliability coefficient: Cronbach’s α = .82) concerns attitude to perseverance, precision, and rationality. These people often like to plan all the aspects of their life and are methodical and precise. “Imagination” (reliability coefficient: Cronbach’s α = .82) involves the attitude to creativity and imagination. “Defensiveness” (reliability coefficient: Cronbach’s α = .79) concerns distrust, rigidity, and materialism. Finally, “introversion” (reliability coefficient: Cronbach’s α = .72) involves introspection and self-isolation. The persons with high scores in this trait often are introvert and can control their instincts. For each trait, the range scores were from 1 to 5 (1 = *very low*, 5 = *very high*).

### Statistical Analyses

First, percentages of the responses to the risk perception questionnaire and *t* test and ANOVA analyses by sociodemographic variables were calculated. Second, with the aim of looking at differences between groups of people, with respect to their personality and self-efficacy, several ANOVA analyses were conducted. The participants were divided in macro groups on the basis of their personality traits and self-efficacy level. The goal of these analyses was to evidence whether people who presented different characteristics had different risk perception for the A/H1N1 virus. Third, several multiple regression analyses were calculated, with the aim to better define the relationships between personality, self-efficacy, and the different aspects of risk perception. Data were analyzed using SPSS package.

## Results

### Risk Perception and Information Sources

All participants referred to know influenza and were conscious it is a transmissible disease. With regard to the A/H1N1 influenza, although more than 65% of the participants thought it was a serious illness, most of them believed that it was unlikely they would develop this disease in the forthcoming flu season, and only 3.2% of the respondents thought they were at risk to get this disease (see Table 2 for details).

**Table 2.** Frequencies and Percentages of the Responses to the “Risk Perception Questionnaire” (*N* = 442).

	Frequency	%	Valid percent	Cumulative percent
Seriousness				
No answer	3	0.9	0.9	0.9
Not serious	43	9.5	9.5	10.4
Middle serious	96	21.7	21.7	32.1
Serious	156	35.3	35.3	67.4
Extremely serious	144	32.6	32.6	100.0
Susceptibility				
No answer	6	1.4	1.4	1.4
Very unlikely	116	26.2	26.2	27.6
Unlikely	164	37.1	37.1	64.7
Not likely/not unlikely	142	32.1	32.1	96.8
Likely	14	3.2	3.2	100
Comparative susceptibility				
No answer	6	1.4	1.4	1.4
Smaller chance	106	24.0	24.0	25.3
Some chance	166	37.6	37.6	62.9
Larger chance	138	31.2	31.2	94.1
Much larger chance	26	5.9	5.9	100.0

### Perceived Risk by Sociodemographic Variables: t Test and ANOVA Analyses

Differences in risk perception by age, gender, education, the status of employment, the size of household, and children aged until 12 years in the household were analyzed (Table 3).

**Table 3.** Means Standard Deviations, and Means Differences Values of the Risk Perception Questionnaire Scores by Sociodemographic and Psychological Variables (Significant Values).

	Risk perception scores								
	Seriousness			Susceptibility			Comparative susceptibility		
	*M*	*SD*	Means differences	*M*	*SD*	Means differences	*M*	*SD*	Means differences
Sociodemographic variables									
Age									
≥1950	2.76	1.04	*t* = 3.33**	2.01	0.846	*t* = −2.39*	2.07	0.940	*t* = −2.48*
<1950	3.26	0.76		2.33	0.80				
Education									
Primary school	3.10	0.94	*F* = 4.04*	2.41	0.86	*F* = 0.016	2.41	0.76	*F* = 2.05
Secondary school	3.14	0.79		1.89	0.88		1.98	1.00	
High secondary school	2.73	1.08		2.02	0.84		2.13	0.90	
University degree	2.54	1.03		2.18	0.81		2.21	0.87	
Psychological variables									
Self-efficacy									
≤25.9	3.10	0.855	*F* = 1.75	2.33	0.887	*F* = 3.54*	2.55	0.783	*F* = 5.01*
25-27.9	3.00	1.00		2.15	0.933		2.05	0.793	
28-30.9	2.85	1.00		2.12	0.859		2.15	0.902	
>31	2.68	1.08		1.81	0.766		1.91	0.987	
Dynamicity									
Very low	3.20	0.88	*F* = 2.46*	2.19	0.798	*F* = 0.493	2.25	0.756	*F* = .1.06
Low	2.83	0.926		2.04	0.893		2.26	0.943	
Middle	2.59	1.09		2.08	0.954		2.19	0.938	
High	2.74	1.01		1.91	0.793		1.83	0.887	
Very high									
Imagination									
Very low	3.17	0.877	*F* = 3.107*	2.19	0.882	*F* = 1.53	2.28	0.825	*F* = 1.66
Low	2.80	0.959		2.09	0.917		2.11	0.945	
Middle	2.83	1.09		2.14	0.879		2.31	0.993	
High	2.62	0.98		1.76	0.819		1.85	0.892	
Very high	2.46	1.33		2.23	0.725		2.15	0.899	
Vulnerability									
Very low	2.55	1.150	*F* = 2.55*	1.91	0.936	*F* = 1.08	2.19	0.892	*F* = 0.47
Low	3.00	1.000		2.16	0.931		2.08	0.929	
Middle	3.00	0.889		2.00	0.827		2.23	0.922	
High	3.12	0.879		2.21	0.709		2.32	0.945	
Very high	2.68	0.945		2.24	0.879				
Conscientiousness									
Very low	2.80	0.980	*F* = 7.38	2.00	0.806	*F* = 3.18*	2.20	0.749	*F* = 3.15*
Low	3.17	1.03		2.21	0.819		2.28	0.922	
Middle	2.82	1.06		2.35	0.889		2.44	0.898	
High	2.89	0.93		1.81	0.761		1.89	0.847	
Very high	2.94	0.98		2.15	0.989		2.00	1.044	

* *p* < .05. **p < .001.

With regard to “age,” the participants were divided into two groups (people born before or after 1950). The choice of this age range depended on the consideration that people born until 1950 could have developed a partial immunity to A/H1N1. These people showed a lower risk of infection during the 2009 pandemic, compared with the other persons, and the media publicized this immunity during the pandemic. The *t* test analysis showed that the older respondents obtained higher risk perception scores compared with the other participants (seriousness: *t* = 3.33, *p* < .001; personal susceptibility: *t* = 2.39, *p* = .02; comparative susceptibility: *t* = 2.48, *p* = .02).

With regard to the “grade of education,” ANOVA showed that the less educated participants obtained higher “risk perception” scores compared with the most educated participants (seriousness: *F* = 4.04, *p* = .008; personal susceptibility: *F* = 3.52, *p* = .016; comparative susceptibility: *F* = 2.05, *p* = .10). The analyses of covariance using the “grade of education” as fixed factor, “age” as a covariate, and the risk perception scores as dependent variables showed that the covariate did not predict the dependent variable.

The “gender,” “status of employment,” “size of the household,” and “children aged <12 years in the household” did not influence risk perception for A/H1N1.

### Risk Perception by Self-Efficacy: ANOVA Analysis

The participants were divided into four groups, based on their GSE scores (first group: from 1° to 25° percentiles; second group: from 26° to 50° percentiles; third group: from 51° to 75° percentiles; fourth group: 76° percentiles and more). The more self-efficacious respondents obtained lower scores of “susceptibility” (*F* = 3.54, *p* = .01) and “comparative susceptibility” (*F* = 5.01, *p* = .002) than the other participants.

### Risk Perception by Personality Traits: ANOVA Analyses

Participants were divided into five groups for each trait, in accordance with the indications of Perussia and Vaino (1996). Several ANOVA analyses, using the level of each trait as the independent variable and the perceived “seriousness,” “personal susceptibility,” and “comparative susceptibility” scores as the dependent variables, were calculated. Results showed that “dynamicity” (*F* = 2.48, *p* = .046), “imagination” (*F* = 3.10, *p* =.01), and “vulnerability” (*F* = 2.52, *p* = .04) influenced the “perceived seriousness” while “conscientiousness” influenced the “personal susceptibility” (*F* = 7.38, *p* = .04) and “comparative susceptibility” (*F* = 3.15, *p* = .015). People who are more conscientious perceived themselves as more susceptible to the A/H1N1 virus than less conscientious people.

### Regression Analyses Using the Perceived “Seriousness,” “Susceptibility,” and “Comparative Susceptibility” as the Dependent Variable

Based on the results of the ANOVA analyses, which showed that people with different characteristics of personality presented different risk perception, several multiple regression analyses were conducted. The first regression analysis used “dynamicity,” “vulnerability,” and “imagination” scores, with control for age and level of education as the independent variables and “perceived “seriousness” as the dependent variable. The second regression analysis used age, level of education, “self-efficacy,” and “conscientiousness” as the independent variables and perceived “susceptibility” as the independent variable. The third regression used age, level of education, “self-efficacy,” and “conscientiousness” as the independent variables and perceived “comparative susceptibility” as the dependent variable.

Results showed that self-efficacy and personality traits were significant predictors of different aspects of risk perception (perceived “seriousness”: *F* = 2.68, *p* = .004, *R*^2^ = .11; “susceptibility”: *F* = 1.89, *p* = .04, *R*^2^ = .10; “comparative susceptibility”: *F* = 2.06, *p* = .02, *R*^2^ = .09). In particular, “imagination” was predictor of the perceived “seriousness” (*t* = −2.14, *p* = .03, Std β = −.17), while “self-efficacy” was predictor of the perceived “susceptibility” (*t* = −3.11, *p* = .002, Std β = −.22) and “comparative susceptibility” (*t* = −2.95, *p* = .003, Std β = −.21; see Table 4).

**Table 4.** Regression Analyses Using the Risk Perception Scores as the Dependent Variable.

	Std β	*t*	Significance
Severity		*F* = 2.68; significance *p* = .004; *R*^2^ = .11	
Age	.01	0.16	.86
Level of education	−.11	−1.46	.14
Dynamicity	−.06	−0.76	.44
Vulnerability	.08	1.21	.22
Imagination	−.17	−2.14	.03*
Susceptibility		*F* = 1.89; significance *p* = .02; *R*^2^ = .10	
Age	.07	1.03	.30
Level of education	−.00	−0.08	.93
Self-efficacy	−.21	−2.95	.003*
Conscientiousness	.04*	0.57	.56
Comparative susceptibility		*F* = 2.06; significance *p* = .02; *R*^2^ = .09	
Age	.12	1.6	.09
Level of education	.04	0.61	.54
Self-efficacy	−.22	−3.22	.001*
Conscientiousness	−.01	−0.21	.83

* *p* < .05.

## Discussion

WHO highlighted the importance for people to protect themselves and their family against infectious pathologies, such as the flu. Among the influenza viruses, the A/H1N1 is the cause of severe cases, and it is expected to remain circulating for many years. Literature (e.g., Guan et al., 2015; Mishra, 2015) reported a wide spread of this virus in recent seasons, with numerous hospitalizations and deaths. Although the vigilance of the health care services for this disease is high, the issues of this study showed that risk perception is low.

The majority of the respondents believed not likely this form of influenza, and the perceived “susceptibility” was low. Perceived “susceptibility” was less than that found in the studies conducted during the pandemic (e.g., Eastwood, Durrheim, Jones, & Butler, 2009; Lau et al., 2009; Seale et al., 2010; Seale et al., 2009). This result could be due to an optimistic bias or a lack of information. However, it is not surprising. After the pandemic, the media attention to this topic declined, and previous studies found a decreasing trend in perceived susceptibility and severity as the number of new infections declined (Barr et al., 2008; Bults et al., 2011; de Zwart et al., 2010; Lau et al., 2010). These findings confirmed this trend.

Moreover, issues of the present study showed that the younger respondents thought that they were at lower risk than the older adults. This belief does not take into account the actual characteristics of the virus. This type of influenza during the pandemic was more frequent in younger people compared with the older adults, and hospitalization rate and mortality among children, teenagers, and younger adults were higher than during seasonal influenza (Louie et al., 2009). The virus continues to cause great complication in middle-aged adults (e.g., Mishra, 2015) and, in some countries, the mean age of hospitalized children with A/H1N1 influenza is lower than that of the peers affected by other flu viruses (Guan et al., 2015). For this characteristic, A/H1N1 influenza should be treated differently from other types of flu, for which older adults are the risk group (Dawood et al., 2009). In this regard, the WHO postpandemic recommendations highlighted that healthy young subjects could continue to be affected disproportionately by severe disease from A/H1N1. The study of Dávila and colleagues (2014) confirmed this trend.

Moreover, the present results also showed that the participants with a low education level believed to be at higher risk, independently of age. This issue is of interest. The most educated people should have access to scientific information about this disease. Despite this, they were not worried about this illness.

The size of the household and the presence of children aged up to 12 years in the household did not influence risk perception of getting A/H1N1. The belief that the presence of children in the family did not affect the risk of a disease is contrary to established scientific evidence. It is known in medicine that flu transmission from children to adults in a household is frequent (Fox, Hall, Cooney, & Foy, 1982; Longini, Koopman, Monto, & Fox, 1982) and the size of the household increases the possibility of contracting the virus. Nevertheless, the likelihood that a person of the family can be the cause of transmission of illness is underestimated. Moreover, children are not considered carriers of infection, although they have a high role in the dissemination of influenza in the household and contribute extensively to the spreading of viruses in the population (e.g., Viboud et al., 2004). Probably parents of children did not take into account that often their sons spend much time in communities, such as school or gym, where the contacts with other children and adults are extensive, with increased risk to get diseases. This unrecognized risk factor, combined with the lack of recommendation for flu vaccination in healthy children, enhances the probability to diffusion of influenza viruses and increases the risk of serious health consequences for specific at-risk groups of people, such as pregnant women.

With regard to the psychological variables, the result showed that the self-efficacious participants felt themselves at lower risk for this disease compared with the other respondents. This finding is of interest because self-efficacy is a predictor of behavior (Bandura, 1998), and it has a regulatory function in different health domains, such as adherence to medical recommendations.

Self-efficacy contributes to preventive behavior, and the adoption of health-promoting behavior depends on individual beliefs of being able to perform behavior appropriately (Bandura, 1997). Persons with high self-efficacy are usually more likely to engage in healthy behaviors, to maintain them, and to recover from setbacks. In this regard, a study (Liao, Cowling, Lam, Ng, & Fielding, 2010) showed that self-efficacy was strongly associated with trust in government/media information on A/H1N1 influenza during the pandemic. Another study, which examined the perceptions and behaviors of people during the early phase of the pandemic in The Netherlands (Bults et al., 2011), demonstrated that high self-efficacy was associated with having a strong intention to comply with government-advised preventive measures in the future and taking protective measures.

Interestingly, in this study, the more self-efficacious respondents did not believe to be at risk for A/H1N1 influenza, and so it is probable that they will not activate protective behavior. Paradoxically, the persons potentially able to comply with government-advised preventive measures did not perceive themselves at risk for this disease.

Findings of this study also showed that people with different personality characteristics presented a different level of risk perception. These issues contributed to clarifying the results of previous studies that demonstrated that various aspects of the personality affected risk perception and engaging in health-protective behavior (Bogg & Roberts, 2004; Deneve & Cooper, 1998; Friedman, 2008; Steel, Schmidt, & Shultz, 2008). In this study, whereas the dynamic and imaginative respondents did not consider the A/H1N1 influenza a serious illness, the conscientious participants perceived themselves at higher risk to get A/H1N1 influenza than the others. The regression analyses contributed to clarifying these results better and showed the importance of “self-efficacy” and “imagination” as significant predictors of risk perception.

All these findings showed the importance that health authorities and media take into account the need to adjust messages of the informative campaigns to different target groups. Health authorities and media should become aware that educational campaigns developed for a particular group of persons could be inadequate for another group. The lack of differentiation of the informative messages during the prevention’s campaign against the pandemic A/H1N1 virus in Italy was probably one of the reasons why people did not understand the actual characteristics of the virus. The educational campaigns during the pandemic in Italy were focused on a character of a cartoon. Indeed, an effective and informative message has to possess structure and characteristics that allow overcoming differences in perceptions associated with the differences in personality profiles.

Personality determines the way people feel and perceive the physical, social, and psychological environment, and for this reason, individuals interpret events diversely and respond differently to the same situation. Health services should take into account that a message could be “read” diversely by different individuals, and educational campaigns should present information in various forms to reach all people. For example, the previously cited Italian campaign with characters of cartoons may not have been considered worthy of attention by some groups of individuals who do not consider a cartoon as an authoritative source of information.

In interpreting the results of the present study, certain limitations should be acknowledged. First, the study is an exploratory research. Moreover, the participants were not representative of the Italian population and not all the variables that contribute to the risks perception are investigated in this study. However, despite these limitations, the issues of the present study could indicate directions for future research, with the aim to plan educational campaigns that consider the complexity of the factors that modulate their efficacy.

## Conclusion

WHO (2015) highlighted the need to investigate people perception of the infectious diseases and assess the ability of the communication channels to disseminate adequate information. The present issues showed a misperception of the risk of the influenza viruses. In particular, the actual characteristic of A/H1N1 virus did not influence risk perception. Subjective variables, such as self-efficacy and personality traits, contributed to determining the perception of the risk. This finding might have implications for the ameliorating public health communication efforts and favorable response to a new influenza outbreak.

For the promotion of precautionary behavior among the populations, health authorities need to know how people perceive risks and whether they will able to use the information on a disease. The evidence that sociodemographic and psychological factors contribute to risk perception shows the need to take into account these variables in the planning of educational campaigns. These variables contribute, in fact, to the personal way the educational messages are received. A better understanding of the role of these variables in risk perception could provide useful information for health risk communication and achieve favorable changes in public behavior, such as WHO (2015) recommends.

Relationships between risk perceptions and social, physical, and psychological variables can supply a key for explaining the difficulty of the success of informative campaigns on health behavior (see, for example, antismoking campaigns) and aid to better define reasons due to which informative and educational campaigns on health often do not reach their expected aims. The evidence of a pivotal role of sociodemographic and psychological variables in determining people ideas and beliefs on a disease and its diffusion highlights the need to carefully design informative campaigns that take into account all the variables that contribute to determining the personal way the informative message is received. This meticulous planning could allow to avoiding that the beliefs about a disease and risk perception depend on personal variables instead of objective variables, such as the actual features of a disease. Health authorities should consider these aspects in their risk communication with the public. Surveillance of perceptions and a better understanding of the role of specific variables in risk perception among the general population will provide useful information for health risk communication and achieve successful changes in public behavior. The future research might better define the role of the contribution of personality and self-efficacy on risk perception, and better investigate how the role of these variables is limited by other concomitant factors, such as previous illness or the habit to vaccination. As Freimuth and Quinn (2004) have highlighted, health communication can increase awareness of a health issue; influence beliefs, attitudes, or behavior; show the benefit of behavior change; and refute myths and misconception. Systematic variations in messages, to increase relevance for different audience segments, should guide planning of the educational campaign.
